# Association of the Dietary Plant-To-Animal Protein Intake Ratio with the Incidence of Slow Gait Speed in Older Adults

**DOI:** 10.1016/j.tjnut.2025.101266

**Published:** 2025-12-09

**Authors:** Emma Huijgen, Hanneke AH Wijnhoven, Marjolein Visser

**Affiliations:** 1 Department of Health Sciences, Faculty of Science, Vrije Universiteit Amsterdam, Amsterdam, the Netherlands; 2 Amsterdam Public Health Research Institute, Amsterdam, the Netherlands

**Keywords:** physical performance, old age, planetary diet, plant-based protein, sustainable diet

## Abstract

**Background:**

Although plant proteins have less environmental impact than animal proteins, it remains unclear whether they can adequately support physical functioning in old age.

**Objective:**

This prospective study aimed to investigate the association of the dietary plant-to-animal protein intake ratio with the incidence of slow gait speed among older adults.

**Methods:**

Data from 997 adults [50.7% male, mean age 65.5 (SD 6.9) y] with a baseline gait speed ≥0.8 m/s were derived from the Longitudinal Aging Study Amsterdam. The dietary plant-to-animal protein intake ratio was calculated from a 238-item food frequency questionnaire completed from 2014 to 2015. Gait speed was measured at baseline and at three 3-y follow-up waves using a 6-meter walk test. Cox proportional hazards models estimated the association between protein ratio quintiles and incident slow gait speed (<0.8 m/s), while adjusting for demographic and lifestyle factors and testing for interaction by sex, overall diet quality, protein intake, and baseline gait speed.

**Results:**

The median dietary plant-to-animal protein intake ratio was 0.67 [interquartile range (IQR): 0.52 to 0.86]. During follow-up, slow gait speed (<0.8 m/s) developed in 415 participants (41.6%). No significant association was found between the protein ratio and incident slow gait speed. The adjusted hazard ratio of the highest (ratio > 0.91) compared with the lowest (ratio ≤ 0.49) quintile was 0.98 (95% confidence interval: 0.68, 1.42; trend across quintiles *P* = 0.89). No significant interactions were observed with sex, overall diet quality, or total protein intake. A higher plant-to-animal protein ratio was suggested to be associated with a lower risk of incident slow gait speed in those with relatively faster baseline gait speed, although associations were not statistically significant.

**Conclusions:**

Among Dutch older adults, the dietary plant-to-animal protein intake ratio was not associated with the risk of developing slow gait speed, suggesting that a more sustainable diet including higher plant protein intake may not compromise physical functioning in older adults.

## Introduction

To maintain independence and a good quality of life, it is important for older adults to preserve their physical functioning. Reduced physical performance, reflected by low values in measures such as gait speed, has been associated with adverse outcomes, including dependency in daily activities, frailty, disability, and mortality among older adults [[1], [2], [3]]. Muscle mass and muscle strength are key factors in maintaining physical performance [4,5].

Adequate nutrition, particularly sufficient protein intake, is important for maintaining muscle mass and physical functioning [6,7]. Dietary protein provides essential amino acids (EAA), which are necessary for muscle protein synthesis and preservation of muscle mass [8]. Aging, however, reduces the muscle’s anabolic response to protein intake, known as anabolic resistance, making older adults more susceptible to muscle loss [9]. For this reason, some experts suggest that older adults may benefit from a higher intake of ≥1.2 g per kg body weight (BW) per day [10,11], although not all studies support this [12,13].

Although total protein intake is important, protein source may also influence muscle health, as different sources vary in protein quality [14]. Protein quality is determined by EAA composition, digestibility, and absorption efficiency [15]. Animal proteins are generally considered superior to plant proteins for muscle growth due to their higher digestibility and complete EAA profile [16,17]. In contrast, many plant proteins are deficient in specific EAAs and less digestible, which may reduce their anabolic potential [[18], [19], [20]]. However, combining different plant-based protein sources can help create a more complete EAA profile [21].

From a sustainability perspective, plant-based foods are advantageous over animal-based foods, as their production generally requires less water, land, and energy [22]. Additionally, greater consumption of plant-based foods has been linked to improved health outcomes, including reduced risks of cardiovascular disease, hypertension, and type 2 diabetes [[23], [24], [25], [26]].

Extensive research has examined sources of protein intake and their short-term effects on muscle mass and strength in older adults. These trials, often focused on isolated protein sources, such as soy or pea protein, rather than the diverse combinations of plant-based proteins, are typically consumed in everyday diets [[27], [28], [29]]. Less emphasis has been placed on the association between the source of protein intake and long-term change in physical functioning. Two earlier cohort studies addressing this topic showed that higher plant protein intakes were associated with less decline in walking performance [30,31]. In contrast, another study showed that higher animal protein intake was associated with less self-reported functional impairment [32]. These studies were predominantly conducted in an Asian population whose dietary patterns differ significantly from those in Western countries, potentially limiting the generalizability of the findings [[30], [31], [32]]. More studies using gait speed, an objective measure of walking performance, strongly predictive of frailty, disability, morbidity, and mortality [3,33,34], are therefore required.

To address this research gap, this study investigated the association between the dietary plant-to-animal protein intake ratio and 10-year incidence of slow gait speed among older adults.

## Methods

### Study design

This study utilized prospective data from the ongoing population-based Longitudinal Aging Study Amsterdam (LASA) and its ancillary Nutrition and Food-related Behavior study, conducted in 2014-2015. LASA is an ongoing cohort study initiated in 1992–1993, including a representative sample of 3107 community-dwelling older adults, aged 55–85 y, from 3 regions of the Netherlands [35]. Refresher cohorts, including adults aged 55–65 years, were added in 2002–2003 (*n* = 1002) and 2012–2013 (*n* = 1023). Follow-up measurement waves take place every 3 y and include a main interview, a self-administered questionnaire, and a medical interview, conducted at participants’ homes by specially trained and intensively supervised interviewers. Details on the sampling and data collection procedures have been described and published elsewhere [35,36]. Ethical approval for the LASA study and the ancillary study was given by the Ethical Review Board of the Vrije University Medical Center, and informed consent was obtained from all respondents.

### Study sample

For this study, the most recent measurement wave conducted before the ancillary study, in which dietary information was collected, was used as the study baseline (2011–2012 for cohorts 1 and 2, and 2012–2013 for cohort 3, a total *n* = 2545). Participants with missing gait speed data at baseline (*n* = 219), which included those who were unable to complete the gait speed test (e.g., due to inability to walk, use of a wheelchair, physical limitations, or incomplete/assisted test completion) (*n* = 121) were excluded. Participants with a gait speed <0.8 m/s at baseline were also excluded (*n* = 794), as they were not at risk of developing slow gait speed during follow-up [37]. Participants who did not participate in the Nutrition and Food-related Behavior ancillary study in 2014–2015, and therefore, had no dietary intake data, were removed from the sample as well (*n* = 453). Further exclusions included those with incomplete responses on the food frequency questionnaire (*n* = 10), and those with implausible energy intake values, based on Willett’s cut-off values (<800 kcal/d or >4000 kcal/d for males, and <500 kcal/d or >3500 kcal/d for females; *n* = 20) [38].

**FIGURE 1:**
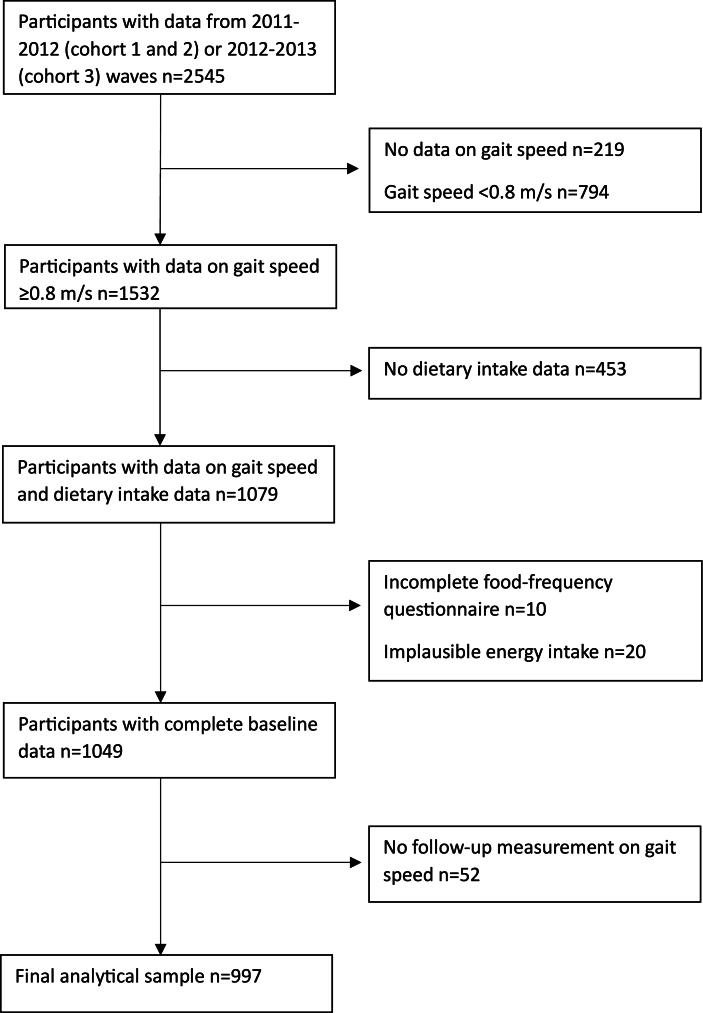
Flowchart describing the selection of the study sample from the Longitudinal Aging Study Amsterdam.

Data from the 2015–2016, 2018–2019, and 2021–2022 measurement waves were used for follow-up. Participants with no follow-up measurement on gait speed, due to loss to follow-up, refusal, or death (0.5%), were excluded from the final analysis (*n* = 52). After applying these exclusion criteria, data from 997 participants remained available for statistical analyses (Figure 1).

### Dietary plant-to-animal protein intake ratio

Dietary intake data were obtained using the Dutch version of the food frequency questionnaire from the Healthy Life in an Urban Setting study. This semiquantitative questionnaire, validated in Dutch older adults, was available in both paper and online formats [39,40]. The questionnaire included 76 questions on food intake over the past 4 wk, covering a total of 238 food items. Each food item was linked to a nutrient database, based on data from the Dutch Food Composition Table 2011, allowing for the calculation of macro- and micronutrient intake [41].

Total protein intake was expressed in grams per day (g/d) and grams per kilogram of BW per day (g/kg BW/d), and was the sum of plant protein and animal protein. Plant proteins refer to proteins obtained from plants, including legumes, seeds and nuts, grains, and vegetables. Animal proteins refer to proteins originating from animal sources, including dairy products, eggs, fish, and meat. The dietary plant-to-animal protein intake ratio was calculated as plant protein intake (g/d) divided by animal protein intake (g/d).

### Gait speed

Gait speed was assessed at baseline and during the follow-up measurement waves. Participants were asked to walk 3 m, make a 180° turn, and walk 3 m back as quickly as possible. Assistive devices were permitted, if needed. A shorter time to complete the walking test reflected better physical performance. A cut-off value of <0.8 m/s was used to define slow gait speed [37].

### Potential confounders

Age (y), sex, baseline gait speed, education level, total energy intake, total protein intake, alcohol intake, diet quality, number of chronic diseases, smoking status, physical activity and BMI (in kg/m^2^) were considered as potential confounders and were a priori selected based on literature suggesting their associations with both (plant- and animal-based) protein intake and physical performance [31,42,43]. Sex, baseline gait speed, diet quality and total protein intake were considered as potential effect modifiers, as previous studies have suggested that the association between protein intake and physical performance may differ by these factors [21,30,[44], [45], [46], [47], [48]]. Data on age and sex were obtained from the baseline interview. During this interview, the highest level of education completed by the participant was obtained and categorized into 3 groups: low (elementary school or less), middle (lower vocational education, general intermediate education, intermediate vocational education, or general secondary education), or high (higher vocational education, college education, or university education). Data on the other variables were obtained during (medical) interviews in 2011–2012 (follow-up measurements for cohorts 1 and 2) and 2012–2013 (baseline measurements for cohort 3). Chronic diseases were self-reported and included the 7 most frequently occurring somatic diseases in the Netherlands: chronic nonspecific lung disease, cardiac disease, peripheral arterial disease, diabetes mellitus, cerebrovascular accident or stroke, osteoarthritis, rheumatoid arthritis, cancer, and other chronic diseases. Smoking status was categorized into never, former, or current smoker. Physical activity was assessed by using the validated LASA Physical Activity Questionnaire, which covers the frequency and duration of activities (such as walking, bicycling, gardening) over the past 2 wk. The intensity of each activity is expressed in Metabolic Equivalent of Task (MET) scores, with a specific MET score assigned to each activity in the questionnaire. A total physical activity score in MET h/wk was calculated by multiplying the frequency, duration, and MET score of each activity [49]. Height was measured to the nearest 0.001 m using a stadiometer and adjusted by subtracting 2.7 cm if the participants wore shoes. BW was measured to the nearest 0.1 kg using a calibrated scale and adjusted by subtracting 1.0 kg if the participants wore clothes. When measured BW was missing, self-reported BW was used. When measured body height was missing, either height from the first follow-up examination wave or self-reported height was used. BMI (kg/m2) categories were defined as: underweight <70 y and <20, ≥70 y and <22; normal and overweight: <70 y and ≥20–30, ≥70 y and ≥22–30, and obese ≥30 [50]. Data on energy intake (kcal/d), total protein intake (g/d), and alcohol intake (g/d) were obtained from the food frequency questionnaire. Diet quality was assessed using the Dutch Healthy Diet Index 2015 (DHD15), which reflects adherence to the Dutch dietary guidelines 2015. The DHD15 score ranged from 0 (lowest adherence) to 130 (highest adherence) [51].

### Statistical analyses

Descriptive statistics were used to summarize the baseline characteristics of the total study sample as well as across quintiles of the dietary plant-to-animal protein intake ratio: first quintile (≤0.49; *n* = 199), second quintile (>0.49 and ≤0.61; *n* = 200), third quintile (>0.61 and ≤0.74; *n* = 199), fourth quintile (>0.74 and ≤0.91; *n* = 200), and fifth quintile (>0.91; *n* = 199). Continuous variables are presented as means and SDs, and categorical variables as frequencies and percentages. Trends in characteristics across the ratio quintiles were tested using linear regression for continuous variables (assigning the median value of each ratio quintile to all participants within that quintile) and using Chi-square tests for categorical variables. Differences in characteristics between older adults developing slow gait speed compared with those not developing slow gait speed were tested using Students’ *t* test for continuous variables and using Chi-square tests for categorical variables.

The event in this analysis was defined as the first occurrence of slow gait speed (<0.8 m/s). Time to event was measured in months, starting from the date of the main interview at baseline to the first occurrence of the event at any follow-up measurement. When a participant was found to have the event at a follow-up measurement after missing the previous follow-up, the date of event was set at the midpoint between the 2 follow-up assessments, as the exact timing of the event was unknown. Participants who died or were lost to follow-up before the occurrence of the event were censored at their last recorded follow-up measurement. Additionally, participants who did not experience the event during any of the follow-up measurements were censored at their final follow-up visit.

To test the association between the plant-to-animal protein ratio and incident slow gait speed, Cox proportional hazards regression models were used. The proportional hazards assumption was assessed by visual inspection of log-minus-log survival curves derived from Kaplan-Meier estimates for each quintile. No deviation from the proportional hazards assumption was detected. The plant-to-animal protein ratio was used as the independent variable, with hazard ratios (HR) and 95% confidence intervals (CI) reported for each quintile, using the first quintile (Q1) as the reference category. *P*-trend analyses were performed to evaluate the linear trend in the incidence of slow gait speed across increasing quintiles of the plant-to-animal protein ratio. To achieve this, a variable was created by assigning the median value of each quintile to all participants within that quintile. Cox proportional hazards regression models were then used to assess associations, with this variable as the independent variable. In addition, a Kaplan-Meier survival analysis was performed to visualize the cumulative incidence of slow gait speed across the quintiles, and a Log Rank test was used to test any difference.

To test potential effect modification of the association between the plant-to-animal protein ratio and the incidence of slow gait speed, interaction terms were included in the unadjusted models for sex, diet quality score (dichotomized into below <83.09 compared with at or above the sample median), total protein intake (dichotomized into not meeting <0.83 g/kg BW/d compared with meeting the Dutch protein intake reference value for older adults), and baseline gait speed [dichotomized into below or at compared with above (>1.0 m/s) the sample median]. Four adjusted Cox proportional hazard regression models were used: model 1, adjusted for demographic factors, including sex, age, and education. Model 2, further adjusted for energy intake and total protein intake (g/kg BW/day). Model 3, further adjusted for the remaining lifestyle factors and health, including alcohol intake, diet quality, smoking status, physical activity score, and the number of chronic diseases. The final model, model 4, included all confounders included in model 3 plus BMI (kg/m2).

All statistical analyses were performed using SPSS Statistics version 28 (IBM SPSS Statistics for Windows, Version 28 Armonk: IBM Corp.). A 2-sided *P* value of <0.05 was considered statistically significant.

## Results

Table 1 shows the baseline characteristics of the 997 participants across quintiles of the dietary plant-to-animal protein intake ratio. Approximately half of the sample was male (50.7%), and the mean age at baseline was 65.5 (SD 6.9) y. Mean baseline gait speed was 1.07 (0.2) m/s. Participants had a mean total protein intake of 82.0 (22.8) g/d, equivalent to 1.1 (0.3) g/kg BW/d. The plant-to-animal protein ratio had a median of 0.67 (interquartile range: 0.52-0.86), with values ranging from 0.18 to 3.69.

**TABLE 1:** Baseline characteristics of the 997 older adults according to quintiles of the dietary plant-to-animal protein intake ratio; the Longitudinal Aging Study Amsterdam^1^.

Participant characteristic	Total sample	By quintile of the dietary plant-to-animal protein intake ratio					*P*-trend
		Q1 = low ≤0.49	Q2 >0.49, ≤0.61	Q3 >0.61, ≤0.74	Q4 >0.74, ≤0.91	Q5 = high >0.91	
N	997	199	200	199	200	199	
Male sex, *n* (%)	505 (50.7)	90 (45.2)	106 (54.5)	97 (48.%)	112 (56.0)	97 (48.)	0.17
Age (y)	65.5 (6.9)	66.3 (7.4)	65.3 (6.7)	65.4 (6.7)	65.5 (6.9)	65.1 (6.5)	0.14
Education^2^							
Low, *n* (%)	93 (9.3)	27 (13.6)	15 (7.5)	18 (9.0)	11 (5.5)	22 (11.1)	0.06
Middle, *n* (%)	568 (57.0)	107 (53.7)	125 (62.5)	136 (68.3)	112 (56.0)	88 (44.2)	
High, *n* (%)	336 (33.7)	65 (32.7)	60 (30.0)	45 (22.6)	77 (38.5)	89 (44.7)	
Energy intake (kcal/d)	2111 (574)	2020 (543)	2149 (571)	2147 (596)	2140 (575)	2098 (578)	0.47
Total protein intake (g/d)	82.0 (22.8)	88.6 (23.9)	85.9 (22.5)	82.1 (22.5)	80.0 (20.8)	73.1 (21.3)	<0.001
Animal protein intake (g/d)	49.1 (17.5)	63.7 (18.0)	55.5 (14.7)	49.3 (13.7)	44.0 (11.6)	32.9 (11.5)	<0.001
Plant protein intake (g/d)	32.9 (10.4)	24.9 (6.9)	30.4 (7.9)	32.8 (8.9)	36.0 (9.4)	40.2 (11.4)	<0.001
Total protein intake (g/kg BW/d) 3	1.1 (0.3)	1.1 (0.3)	1.1 (0.3)	1.1 (0.3)	1.0 (0.3)	1.0 (0.3)	<0.001
Alcohol intake (g/d)	15.0 (17.1)	17.7 (19.6)	15.4 (15.2)	14.9 (19.7)	14.8 (16.0)	12.5 (13.9)	0.004
Diet quality, DHD15-index score^4^	83.2 (16.1)	76.1 (15.1)	79.7 (16.3)	84.0 (15.9)	86.8 (14.2)	89.3 (15.5)	<0.001
No. of chronic diseases	1.0 (0.8)	1.0 (0.8)	0.9 (0.8)	0.9 (0.8)	0.8 (0.8)	0.8 (0.8)	0.012
No chronic disease, *n* (%)	362 (36.3)	62 (31.2)	71 (35.5)	62 (31.2)	87 (43.5)	80 (40.2)	0.03
One chronic disease, *n* (%)	396 (39.6)	78 (39.2)	83 (41.5)	85 (42.7)	71 (35.5)	78 (39.2)	
≥2 chronic diseases	240 (24.1)	59 (29.6)	46 (23.0)	52 (26.1)	42 (21.0)	41 (20.6)	
Smoking status^3^							
Current, *n* (%)	101 (10.5)	23 (11.9)	26 (13.1)	22 (11.6)	17 (8.%)	13 (6.9)	0.26
Former, *n* (%)	596 (61.8)	116 (60.1)	121 (60.8)	119 (62.2)	126 (64.9)	114 (60.6)	
Never, *n* (%)	267 (27.7)	54 (28.0)	52 (26.1)	49 (25.8)	51 (26.3)	61 (32.4)	
Physical activity (MET h/wk)^5^	63.8 (42.3)	61.5 (41.1)	64.5 (49.1)	63.4 (37.6)	66.2 (45.9)	63.4 (36.9)	0.70
BMI (kg/m2)^3^	26.9 (4.1)	27.8 (3.9)	27.6 (4.4)	27.0 (3.9)	26.4 (3.6)	25.4 (3.9)	<0.001
Underweight, *n* (%)^6^	24 (2.5)	2 (1.0)	7 (3.5)	4 (2.1)	5 (2.6)	6 (3.2)	0.003
Normal weight or overweight, *n* (%)	758 (78.6)	144 (74.6)	142 (71.4)	149 (78.8)	160 (82.5)	163 (86.2)	
Obese, *n* (%)	182 (18.9)	47 (24.4)	50 (25.1)	36 (19.0)	29 (14.9)	20 (10.6)	
Baseline gait speed (m/s)	1.1 (0.2)	1.1 (0.2)	1.1 (0.2)	1.1 (0.2)	1.1 (0.2)	1.1 (0.2)	0.23

Abbreviations: DHD15, Dutch Healthy Diet Index 2015; MET, Metabolic Equivalent of Task.

1 Categorical data are presented as *N* (percentage) and continuous data as mean (standard deviation).

2 Education level low (elementary school or less), middle (lower vocational education, general intermediate education, intermediate vocational education, or general secondary education), or high (higher vocational education, college education, or university education).

3 Total protein intake per kilogram body weight, smoking status, and BMI were based on 964 participants.

4 DHD15 range 0 (lowest adherence) to 130 (highest adherence).

5 Physical activity was based on 996 participants.

6 Underweight Underweight <70 y <20 kg/m^2^, ≥70 y <22 kg/m^2^, normal or overweight: <70 y ≥20–30 kg/m^2^, ≥70 y ≥22–30 kg/m^2^, and obese ≥30 kg/m^2^

Participants with a higher proportion of protein intake from plant sources were more likely to have a higher education level and had a healthier lifestyle (e.g., lower prevalence of current smoking, a lower alcohol intake, and a higher diet quality score). They also had lower total daily protein intake, lower BMI (kg/m2), and fewer chronic diseases compared with those with a lower proportion of protein intake from plant sources.

**FIGURE 2:**
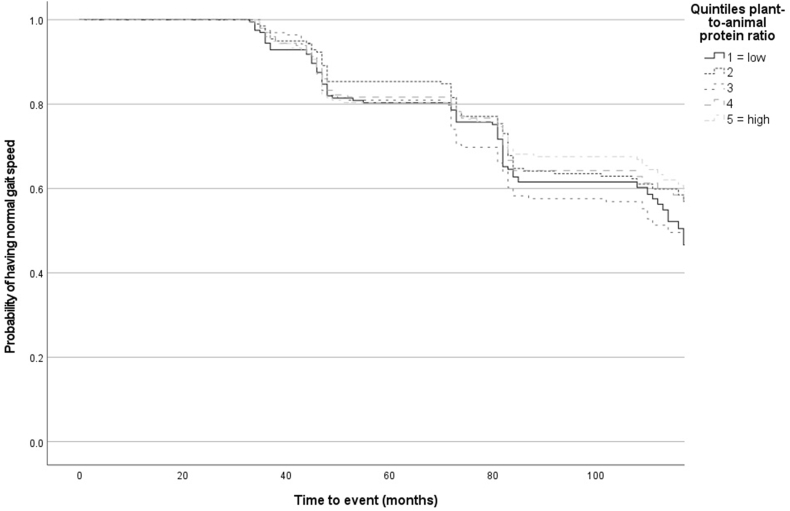
Kaplan-Meier curves for maintaining normal gait speed (≥0.8 m/s) according to quintiles of the dietary plant-to-animal protein ratio in 997 older adults; the Longitudinal Aging Study Amsterdam^1^ ^1^Curves were truncated after t = 111 mo to avoid misrepresentation due to low numbers at risk and disproportionately low probabilities.

Over a mean follow-up time of 86.6 mo (range: 32–127 mo), 415 participants (41.6%) developed slow gait speed (<0.8 m/s). Participants who developed a slow gait speed during follow-up were older, more likely to be male, more likely to be current smokers, had a lower education level, a lower alcohol intake, a higher BMI (kg/m2), more chronic diseases, and were less physically active (Table 2). The Kaplan-Meier curves in Figure 2 show no significant difference in the risk of slow gait speed development across the quintiles of the plant-to-animal protein ratio (Log Rank test, χ^2^ (4) = 4.50, *P* = 0.343).

**TABLE 2:** Baseline characteristics of the 997 older adults according to developing or not developing slow gait speed (<0.8 m/s) during follow-up; the Longitudinal Aging Study Amsterdam^1^.

Participant characteristic	Developing slow gait speed	Not developing slow gait speed	*P* value
*N*, *n* (%)	415 (41.6)	582 (58.4)	
Male sex, *n* (%)	195 (47.0)	310 (53.3)	0.051
Age (y)	63.6 (5.5)	68.2 (7.6)	<0.001
Education^2^			
Low, *n* (%)	54 (13.0)	39 (6.7)	<0.001
Middle, *n* (%)	262 (63.1)	306 (52.6)	
High, *n* (%)	99 (23.9)	237 (40.7)	
Energy intake (kcal/d)	2117.6 (590.7)	2106.1 (562.1)	0.76
Total protein intake (g/d)	82.3 (23.0)	81.7 (22.7)	0.71
Animal protein intake (g/d)	50.0 (17.7)	48.5 (17.4)	0.19
Plant protein intake (g/d)	32.3 (10.1)	33.3 (10.5)	0.16
Total protein intake (g/kg BW/d)^3^	1.0 (0.3)	1.1 (0.3)	0.54
Alcohol intake (g/d)	13.5 (16.3)	16.1 (17.6)	0.02
Diet quality, DHD15-index score^4^	82.7 (16.0)	83.5 (16.2)	0.41
No. of chronic diseases	1.1 (0.8)	0.7 (0.7)	<0.001
No chronic disease, *n* (%)	113 (27.2)	249 (42.8)	<0.001
One chronic disease, *n* (%)	165 (39.8)	230 (39.5)	
Two or more chronic diseases, *n* (%)	137 (33.0)	103 (17.7)	
Smoking status^3^			
Current, *n* (%)	51 (12.6)	50 (8.9)	0.07
Former, *n* (%)	234 (57.8)	362 (64.8)	
Never, *n* (%)	120 (29.%)	147 (26.3)	
Physical activity (MET hours/week)^5^	60.9 (37.9)	65.9 (45.2)	0.06
BMI (kg/m2)^3^	27.4 (4.1)	26.4 (4.0)	<0.001
Underweight^6^, *n* (%)	7 (1.7)	17 (3.0)	0.005
Normal weight or overweight, *n* (%)	304 (75.2)	454 (81.1)	
Obese, *n* (%)	93 (23.0)	89 (15.9)	
Baseline gait speed (m/s)	1.0 (0.2)	1.1 (0.2)	<0.001

Abbreviations: DHD15, Dutch Healthy Diet Index 2015; MET, Metabolic Equivalent of Task.

1 Categorical data are presented as *N* (percentage) and continuous data as mean (standard deviation).

2 Education level low (elementary school or less), middle (lower vocational education, general intermediate education, intermediate vocational education, or general secondary education), or high (higher vocational education, college education, or university education).

3 Total protein intake per kilogram body weight, smoking status, and BMI were based on 964 participants.

4 DHD15 range 0 (lowest adherence) to 130 (highest adherence).

5 Physical activity was based on 996 participants.

6 Underweight <70 y <20 kg/m^2^, ≥70 years <22 kg/m^2^, normal or overweight: <70 y ≥20–30 kg/m^2^, ≥70 y ≥22–30 kg/m^2^, and obese ≥30 kg/m^2^.

Table 3 presents the HRs for incident slow gait speed across quintiles of the dietary plant-to-animal protein intake ratios. In the crude model, participants in the highest quintile (Q5) had a lower, although not statistically significant, risk of developing slow gait speed compared with those in Q1, with an HR of 0.80 (95% CI: 0.58, 1.08). No consistent trend was observed across the quintiles (*P-*trend = 0.14). Adjustment for potential confounders in the different models slightly attenuated the HRs of those in Q5. No trends across the quintiles were observed in the different models.

**TABLE 3:** Risk (hazard ratio with 95% confidence interval) of developing slow gait speed (<0.8 m/s) during follow-up according to quintiles of the dietary plant-to-animal protein intake ratio in 997 older adults; the Longitudinal Aging Study Amsterdam.

	Quintiles of dietary plant-to-animal protein intake ratio					
	Q1 = low	Q2	Q3	Q4	Q5 = high	
Ratio quintile range	≤0.49	>0.49, ≤0.61	>0.61, ≤0.74	>0.74, ≤0.91	>0.91	
*N* study sample	199	200	199	200	199	
Total person months follow-up	17,088	17,822	16,352	17,471	17,609	
Mean follow-up time (months, min-max)	86 (33–127)	89 (33–122)	82 (34–127)	87 (32–127)	88 (34–126)	
No. of events, *n* (%)	88 (44.2)	83 (41.5)	89 (44.7)	81 (40.5)	74 (37.2)	

	HR (95% CI)					*P*-trend

Crude model	1.0	0.89 (0.66–1.21)	1.07 (0.80–1.44)	0.89 (0.66–1.20)	0.80 (0.58–1.08)	0.14
Model 1^1^	1.0	0.91 (0.68–1.24)	1.07 (0.79–1.44)	0.98 (0.72–1.33)	0.89 (0.65–1.21)	0.53
Model 2	1.0	0.87 (0.64–1.19)	1.03 (0.76–1.40)	0.95 (0.70–1.30)	0.83 (0.60–1.15)	0.36
Model 3	1.0	0.87 (0.63–1.19)	1.02 (0.74–1.40)	1.00 (0.72–1.38)	0.82 (0.58–1.15)	0.38
Model 4	1.0	0.92 (0.67–1.27)	1.13 (0.82–1.57)	1.15 (0.81–1.62)	0.98 (0.68–1.42)	0.89

Abbreviations: CI, confidence interval; HR, hazard ratio; NA, not applicable.

1 Model 1 was adjusted for sex, age, and education level; Model 2 was additionally adjusted for energy intake and total protein intake (g/kg BW/day) based on 964 participants; Model 3 was additionally adjusted for alcohol intake, diet quality, physical activity score, number of chronic diseases, and smoking status based on 961 participants; Model 4 was additionally adjusted for BMI based on 961 participants.

**FIGURE 3:**
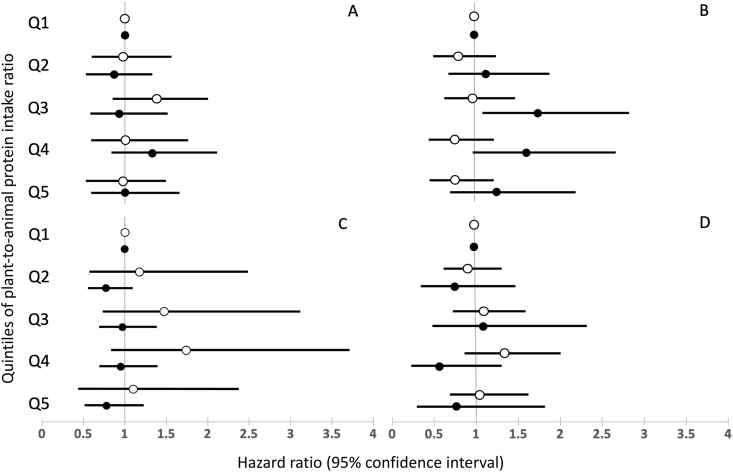
Risk (hazard ratio with 95% confidence interval) of developing slow gait speed (<0.8 m/s) during follow-up according to quintiles of the dietary plant-to-animal protein intake ratio; stratified by sex [men vs. women, (A) P value for interaction was 0.37 for Q2, 0.39 for Q3, 0.07 for Q4 and 0.10 for Q5], baseline dietary quality index score (below sample median <83.09 versus at or above sample median; B) P value for interaction was 0.24 for Q2, 0.44 for Q3, 0.14 for Q4 and 0.12 for Q5), baseline protein intake (not meeting protein intake reference value of 0.83 g/kg BW/d versus meeting reference value, (C) *P* value for interaction was 0.78 for Q2, 0.86 for Q3, 0.48 for Q4 and 0.23 for Q5) and baseline gait speed (below sample median ≤1.0 m/s versus above sample median, D, P for interaction were <0.001 for Q2 to Q5) in older adults; the Longitudinal Aging Study Amsterdam.

The association between the dietary plant-to-animal protein intake ratio and incident slow gait speed did not differ significantly between males and females (*P*-interaction was 0.37 for Q2, 0.39 for Q3, 0.07 for Q4, and 0.10 for Q5) (Figure 3A). The association was also similar for those with a relatively low dietary quality compared to those with a relatively higher diet quality (*P*-interaction was 0.24 for Q2, 0.44 for Q3, 0.14 for Q4, and 0.12 for Q5) (Figure 3B), and for those not meeting or meeting the protein intake reference value (*P*-interaction was 0.78 for Q2, 0.86 for Q3, 0.48 for Q4, and 0.23 for Q5) (Figure 3C).

Significant interaction terms were found between the dietary plant-to-animal protein intake ratio and baseline gait speed (*P*-interaction was <0.001 for Q2 to Q5 of the dietary plant-to-animal protein intake ratio). Stratified analyses were performed, differentiating participants with a faster than the sample median baseline gait speed (>1.0 m/s) and participants with a slower baseline gait speed (≤1.0 m/s) (Figure 3D). Among participants with a relatively faster baseline gait speed, the HR (95% CI) for slow gait speed in each quintile was generally <1.0, as compared to those in Q1 in the fully adjusted model. However, no trend across the quintiles was observed (*P* = 0.39). In contrast, among participants with a slower baseline gait speed, the HR (95% CI) for slow gait speed in each quintile was generally higher than 1.0 compared with those in Q1 in the fully adjusted model, with no trend across the quintiles (*P* = 0.35).

## Discussion

This prospective cohort study investigated the association between the dietary plant-to-animal protein intake ratio and the incidence of slow gait speed during a 10-y follow-up among older adults. No significant differences in the incidence of slow gait speed across the ratio quintiles were observed. The associations were similar between males and females, and did not depend on total protein intake or overall diet quality. A higher plant-to-animal protein ratio was suggested to be associated with a lower risk of incident slow gait speed in those with relatively faster baseline gait speed, although associations were not statistically significant.

Greater consumption of plant-based foods has been linked to improved health outcomes, as well as being more sustainable for the environment. However, traditionally, animal protein has been considered superior due to its higher leucine content, higher digestibility, and complete EAA profile, all of which support muscle protein synthesis [16,17,52]. The assumption that diets high in animal protein are more beneficial for muscle mass and physical functioning has recently been questioned. Diets high in plant proteins are typically rich in potassium and magnesium, and have an alkaline property [53,54], which may support the maintenance of muscle mass and physical functioning in older adults, particularly when protein intake is sufficient, and plant protein sources are diversified to ensure EAA adequacy [54].

Three prospective studies have previously reported on the association between protein source and physical functioning. The first involved 1896 participants aged ≥50 y from the Framingham Offspring Study with a mean follow-up of 14.4 y [32]. Higher animal protein intake was more strongly associated with lower risks of self-reported functional impairment than plant protein intake. However, the study did not adjust for total protein intake, which may have confounded the findings, particularly because participants consuming more animal protein also tended to have higher overall protein intake. Overall dietary quality was also not considered. The other 2 studies were conducted in the same cohort of ∼3000 Chinese community-dwelling adults aged ≥65 y in Hong Kong, who were followed for 4 y [30,31]. Li et al. [30] reported that higher baseline plant protein intake was associated with significantly less decline in gait speed in individuals without sarcopenia (83% of sample). However, the ratio of animal-to-plant protein was not associated with change in gait speed. Yeung and Woo [31] reported that higher plant protein intake was associated with less decline in physical performance, measured by a 6-m walk test in females, but not in males. Both studies expressed plant protein intake in g/kg BW/d and adjusted for total protein intake and energy intake. Similar to the current study, Yeung and Woo [31] additionally adjusted for overall diet quality, whereas Li et al. [30] adjusted for a dietary inflammatory index.

The findings from the 2 studies from the Chinese cohort suggest that higher plant protein intake may potentially be beneficial for maintaining physical performance in older adults, which contradicts the findings of the current study. The average protein intake in the current study was relatively high (on mean 1.1 g/kg BW/d) and may have ensured EAA sufficiency across the cohort, potentially masking any differential associations of protein source. However, no effect modification was observed by total protein intake, contrasting those meeting the protein reference value of 0.83 g/kg BW/d with those not meeting this value. Furthermore, the total protein intake in the Chinese cohort was even higher (mean 1.3 g protein/kg BW/d). Thus, differences in total protein intake are unlikely to explain the contrasting findings.

In the current study, participants in the highest quintile of the plant-to-animal protein ratio still consumed considerable amounts of animal protein. Only a small fraction (4.5%) of this group derived <70% of their total protein intake from plant sources, a level above which EAA deficiencies may become more likely [55]. The limited contrast in protein source composition across quintiles may have reduced the ability to detect associations based on protein source alone. In contrast, populations with a higher proportion of plant-based protein intake may still meet EAA requirements, while also benefiting from the potential advantages of diets higher in plant proteins. Li et al. [30] reported higher average plant protein intakes, with an animal-to-plant protein ratio of 1.24 among participants without sarcopenia, compared with 1.49 in the current study. Similarly, Yeung and Woo [31] reported that females consumed on average 48.7% of their protein from plant sources, compared with 40.8% in the current study. These comparisons suggest that the Chinese cohort consumed significantly more plant protein, which may explain their observed positive association with physical performance. Another potentially important explanation lies in the type of plant protein consumed. Compared with Western populations, Chinese populations generally consume higher amounts of soy protein. Given that soy protein is recognized as a high-quality protein source, with an EAA profile almost equivalent to that of animal proteins, a greater intake may increase the likelihood of observing physical performance benefits associated with a higher plant protein intake [56].

The current study found no sex difference in the association, although this was observed in the study by Yeung and Woo [31]. This discrepancy may partly reflect differences in dietary patterns, as females in the Chinese cohort consumed a greater proportion of plant proteins than males (48.7% compared with 44.0%). In the current study, no such sex differences in protein intakes were observed (∼41% of protein from plant-based sources in both males and females). These observations suggest that the previously observed sex-specific associations may reflect sex-specific differences in dietary patterns rather than physiological differences in response to plant protein.

In the current study, a nonsignificant but suggestive association between a higher plant-to-animal protein ratio and lower risk of incident slow gait speed was observed in participants with a baseline gait speed >1.0 m/s. This observation appears to align with findings from Li et al. [30], who reported a positive association of greater plant protein intake and physical functioning among individuals without sarcopenia (mean baseline gait speed 1.04 m/s), but not in those with sarcopenia (mean baseline gait speed 0.98 m/s). Further studies are needed to determine whether individuals with better baseline physical functioning could potentially be more likely to experience benefits from a higher plant-to-animal protein ratio.

This study has several strengths, which include the long follow-up period and the objective measurement of gait speed, thereby minimizing the risk of subjective bias as observed in self-reported function measures. Dietary intake was assessed using a validated food frequency questionnaire, which enabled estimation of usual protein intake from a variety of sources. Additionally, several potential confounding factors were accounted for in the analyses, including total protein intake and overall diet quality. This adjustment was particularly important, as participants in the highest quintile of the plant-to-animal protein ratio also had the highest diet quality score. However, several limitations must also be acknowledged. First, the study sample consisted exclusively of individuals residing in the Netherlands, which limits the generalizability of the findings to other Western populations with different dietary habits, including those with higher plant-based protein intakes. Second, as individuals with a gait speed <0.8 m/s were excluded, the findings may not be applicable to the frailest segment of the older population. This exclusion may also have reduced the variability in protein (source) intake, reducing the likelihood of observing associations. Third, information on the type of plant proteins was not available. However, in the Netherlands, adults aged 65 to 79 y obtain 51% of their plant protein from cereals and cereal products, 12% from fruits, nuts, seeds, and olives, 10% from vegetables, 6% from potatoes and other tubers, and 6% from cakes and sweet biscuits. Fourth, dietary intake was measured only once, and therefore, potential changes in diet over time were not captured. Furthermore, a food frequency questionnaire relies on memory, which can lead to reporting bias, and may be less accurate in assessing portion sizes. The method can also lead to exposure misclassification, although this may be nondifferential. Finally, as this was an observational study, causal relationships cannot be established.

In conclusion, in this longitudinal study of older adults in the Netherlands, no association was observed between dietary plant-to-animal protein intake ratio and incidence of slow gait speed, even after taking total protein intake and overall diet quality into account. Results were similar for males and females. These findings suggest that consuming a higher proportion of plant protein in the diet does not increase the risk of gait speed decline and may offer a viable and more sustainable dietary pattern for Dutch older adults.

## Author contributions

The authors’ responsibilities were as follows – MV: designed the study and was involved in the data collection; EH, HW prepared the databases; EH: performed the statistical analyses; EH, MV: wrote the paper; and all authors: have read and approved the final manuscript.

## Data availability

The data underlying this article cannot be shared publicly due to the privacy of individuals who participated in the study. The data will be shared on reasonable request to the LASA cohort (https://lasa-vu.nl).

## Funding

The Longitudinal Aging Study Amsterdam is supported by a grant from the Netherlands Ministry of Health, Welfare and Sport, Directorate of Long-Term Care. The data collection in 2012-2013 was financially supported by the Netherlands Organization for Scientific Research (NWO) in the framework of the project “New Cohorts of young old in the 21st century” [file number 480-10-014]. Funding for the Nutrition and Food-related Behavior ancillary study of the Longitudinal Aging Study Amsterdam was provided by the European Union FP7 MooDFOOD Project “Multi-country collaborative project on the role of Diet, Food-related behavior, and Obesity in the prevention of Depression” [grant agreement no. 613598]. These sources had no role in study design; collection, analysis, and interpretation of data; nor in writing of the report.

## Conflict of interest

The authors report no conflicts of interest.
